# Long-term observation of estimated fluid volume reduction after the initiation of ipragliflozin in patients with type 2 diabetes mellitus: a sub-analysis from a randomized controlled trial (PROTECT)

**DOI:** 10.1186/s13098-023-01129-3

**Published:** 2023-07-07

**Authors:** Atsushi Tanaka, Takumi Imai, Shigeru Toyoda, Kazuhiro Sugimoto, Ruka Yoshida, Machi Furuta, Koichi Node, the PROTECT Investigators

**Affiliations:** 1 Department of Cardiovascular Medicine, Saga University, 5-5-1 Nabeshima, Saga, 849-8501 Japan; 2 Department of Medical Statistics, Osaka Metropolitan University Graduate School of Medicine, Osaka, Japan; 3 Department of Cardiovascular Medicine, Dokkyo Medical University School of Medicine, Mibu, Japan; 4 Diabetes Center, Ohta Nishinouchi Hospital, Koriyama, Japan; 5 Department of Cardiology, Japanese Red Cross Aichi Medical Center Nagoya Daini Hospital, Nagoya, Japan; 6 Department of Clinical Laboratory Medicine, Wakayama Medical University, Wakayama, Japan

**Keywords:** Estimated fluid volume, Sodium-glucose co-transporter 2 inhibitor, Ipragliflozin, Type 2 diabetes mellitus, N-terminal pro-brain natriuretic peptide

## Abstract

**Backgrounds/Aim:**

Recent studies have shown that the addition of sodium-glucose co-transporter 2 (SGLT2) inhibitors gradually reduces the estimated fluid volume parameters in a broad range of patient populations, suggesting that this mediates the clinical benefits of SGLT2 inhibitors in preventing heart failure. Here, we sought to examine the long-term (24 months) effect of the SGLT2 inhibitor ipragliflozin on the estimated fluid volume parameters in patients with type 2 diabetes mellitus (T2DM).

**Methods:**

In this prespecified sub-analysis of the PROTECT (Prevention of Atherosclerosis by SGLT2 Inhibitor: Multicenter, Randomized Controlled Study) trial, which was an investigator-initiated, multicenter, prospective, randomized, open-label, clinical trial primarily designed to evaluate the effect of ipragliflozin treatment administered for 24 months on carotid atherosclerosis in patients with T2DM, we evaluated serial changes in estimated plasma volume (ePV, %) calculated using the Straus formula and estimated extracellular volume (eEV, mL) calculated by the body surface area by 24 months following the initiation of 50-mg ipragliflozin once daily and compared them with those following standard care for T2DM (non-SGLT2 inhibitor use).

**Results:**

This sub-analysis included 464 patients (ipragliflozin, *n* = 232; control, *n* = 232), a full analysis set of the PROTECT trial. In an analysis using mixed-effects models for repeated measures, relative to the control group, ipragliflozin significantly reduced ePV by − 10.29% (95% confidence interval [CI]  − 12.47% to − 8.11%; *P* < 0.001) at 12 months and − 10.76% (95% CI − 12.86% to − 8.67%; *P* < 0.001) at 24 months. Additionally, ipragliflozin significantly reduced eEV by − 190.44 mL (95% CI − 249.09 to − 131.79 mL; *P* < 0.001) at 12 months and − 176.90 mL (95% CI  − 233.36 to − 120.44 mL; *P* < 0.001) at 24 months. The effects of ipragliflozin on these parameters over 24 months were mostly consistent across various patient clinical characteristics.

**Conclusions:**

This prespecified sub-analysis from the PROTECT trial demonstrated that ipragliflozin treatment, compared with the standard care for T2DM, reduced two types of estimated fluid volume parameters in patients with T2DM, and the effect was maintained for 24 months. Our findings suggest that SGLT2 inhibitor treatment regulates clinical parameters incorporated into the calculating formulas analyzed and consequently fluid volume status for the long-term, and this may be at least partly associated with clinical benefits from chronic use of SGLT2 inhibitors.

*Trial registration* Japan Registry of Clinical Trials, ID jRCT1071220089

**Supplementary Information:**

The online version contains supplementary material available at 10.1186/s13098-023-01129-3.

## Introduction

The inhibition of sodium-glucose co-transporter 2 (SGLT2) located at the renal proximal tubules essentially increases the urinary excretion of glucose and sodium, thereby immediately promoting osmotic and natriuretic diuresis [1, 2]. Particularly, SGLT2 inhibitors uniquely promote an electrolyte-free water clearance and a greater removal of interstitial fluid volume than circulating volume [3, 4]. This reduces not only cardiac overload but also excess fluid volumes without intravascular volume depletion and compensated sympathetic nerve activation, potentially leading to a maintenance of favorable fluid homeostasis and subsequent cardiorenal benefits [5]. Accordingly, these diuretic actions are likely to explain the primary mechanisms underlying accumulated evidence on SGLT2 inhibitor-induced risk reduction of heart failure (HF) and renal events in a broad range of subjects, irrespective of diabetes and HF clinical situations [6–8]. Thus, the appropriate monitoring of fluid volume status after the initiation of SGLT2 inhibitor therapy may be clinically useful in predicting the cardiorenal benefits of this therapy [9].

Several clinical studies investigating the short- to intermediate-term effects of SGLT2 inhibitor therapy on estimated fluid volume parameters have previously demonstrated a gradual reduction in those parameters following SGLT2 inhibitor administration, which was maintained for several weeks in patients with type 2 diabetes mellitus (T2DM) or HF [10–15]. However, little is currently known about the long-term effects of SGLT2 inhibitor therapy on the estimated fluid volume status. Although an increase in the urine volume is generally transient after the initiation of SGLT2 inhibitor therapy [10, 16], the chronic inhibition of SGLT2 may favorably alter the hemodynamic status via several mechanisms, such as improved cardiovascular function and enhanced erythropoiesis [17–19], followed by long-term cardiorenal benefits. Given the fine prognostic values of estimated fluid volume parameters [20–22], the long-term impact of SGLT2 inhibitors on those parameters merits investigation to clarify their hemodynamic modulations and the clinical usefulness of monitoring them as surrogate markers of cardiorenal benefits in the chronic use of SGLT2 inhibitors. In this sub-analysis from the randomized controlled trial PROTECT (Prevention of Atherosclerosis by SGLT2 Inhibitor: Multicenter, Randomized Controlled Study) for patients with T2DM [23, 24], we sought to examine the effects of the use of the SGLT2 inhibitor ipragliflozin for 24 months on the estimated fluid volume parameters obtained annually for 2 years.

## Methods

### Study design and population

The study was a prespecified sub-analysis of the PROTECT trial (UMIN000018440). The details of the study design, eligibility criteria, and main results have been reported elsewhere [23, 24]. In brief, the PROTECT trial was an investigator-initiated, multicenter, prospective, randomized, open-label, and blinded-endpoint clinical trial conducted in 39 centers in Japan, in which the effect of the use of ipragliflozin for 24 months on carotid intima-media thickness (IMT) was primarily examined in patients with T2DM (HbA1c of 6.0%–10.0% despite diet and exercise therapy and/or standard diabetes medications for at least 3 months before enrollment). The exclusion criteria were patients with severe renal dysfunction (estimated glomerular filtration rate [eGFR] < 45 mL/min/1.73 m^2^), those with a history of cardiocerebrovascular diseases within 3 months before the study, and those with HF with New York Heart Association functional classifications III and IV.

All candidate subjects received a detailed explanation of the study plan and provided written informed consent before enrollment in the study. Eligible subjects were equally randomized to either the group receiving add-on ipragliflozin (50 mg daily) or the control group (non-SGLT2 inhibitor use and continued their background therapy and medications for T2DM), using a web-based modified minimization method balanced for age (< 65 and ≥ 65 years), HbA1c level (< 7.0% and ≥ 7.0%), systolic blood pressure (< 135 and ≥ 135 mmHg), the use of statins, and the use of metformin at the time of screening. Then, the participants were followed up for 24 months after the initiation of the study protocol. During the study protocol, there was a requirement that the background therapy remained, in principle and if possible, unchanged based on the participants’ medical condition.

This prespecified sub-analysis was performed after the publication of the main results of the PROTECT trial [24]. The protocol of a series of PROTECT secondary analyses, including the current analysis, were approved by the Ethics Committee of Saga University Hospital and subsequently registered to the Japan Registry of Clinical Trials (ID jRCT1071220089).

### Study endpoints

The main endpoints in this sub-analysis were the percentage change in the estimated plasma volume (ePV) and the absolute change in the estimated extracellular volume (eEV) from baseline to 12 and 24 months of post-randomization follow-up visits. In the ipragliflozin arm, the correlations between changes in the estimated fluid volume parameters (ePV and eEV) and N-terminal pro-brain natriuretic peptide (NT-proBNP) concentrations from baseline to 24 months after the initiation of ipragliflozin treatment were also examined.

### Estimated fluid volume calculation

The detailed formulas for calculating the estimated fluid volume parameters (ePV and eEV) have been described previously [14, 15]. Briefly, the percentage changes in ePV at each visit after the initiation of the study protocol were calculated using the Strauss formula [11, 12], and the ePV at baseline was calculated using the Kaplan–Hakim formula [25], as follows:

$$\text{Strauss formula: 100}\times \frac{\text{hemoglobin }(\text{at baseline})}{\text{hemoglobin }(\text{at visit})} \times \frac{1-\text{hematocrit }(\text{at visit})}{1-\text{hematocrit }(\text{at baseline})}-{100}$$

$${\text{Kaplan}{-}\text{Hakim formula}}{:}\,(1-\text{hematocrit})\times (a+[b\times\text{body weight}(\text{kg})])$$

where a = 1530 in men and 864 in women, and b = 41 in men and 47.9 in women.

The eEV at each visit, including baseline, was estimated using the following formula [12]:

$$8116.6\times [0.007184 \times \text{height} {\left(\text{cm}\right)}^{0.725}\times \text{weight} {\left(\text{kg}\right)}^{0.425}]-28.2$$

### Statistical analysis

Summary statistics for the baseline demographics and characteristics are expressed as medians (interquartile ranges) for continuous variables and frequencies (%) for categorical data. The mean changes in ePV and eEV from baseline and their 95% confidence intervals (CI) were estimated using mixed-effects models for repeated measures. The effects of ipragliflozin, compared with the control group, on the ePV and eEV over 24 months after the initiation of the study protocol were examined in the entire population and subgroups according to several background information—age, sex, body mass index (BMI) (25 kg/m^2^), eGFR (60 mL/min/1.73m^2^), T2DM duration (10 years), HbA1c level (7.0%), previous disease history (i.e., hypertension, atherosclerotic cardiovascular disease [ASCVD], and HF), and medication use (i.e., statin, metformin, dipeptidyl peptidase-4 inhibitor, and diuretic) and corresponding values at baseline. The NT-proBNP concentration at 24 months was analyzed on its logarithmic scale using a linear regression model, and the proportional changes from baseline to 24 months for both groups were estimated and compared by group ratio. Pearson correlation analyses were performed for the ipragliflozin group to assess the associations between changes from baseline to 24 months in each estimated fluid volume parameter and log-scaled NT-proBNP concentration. All statistical analyses were performed using R (version 4.2.0; R Core Team, 2022). A two-sided significance level of P < 0.05 was used for all assessments, and no adjustment for multiplicity was considered in these analyses.

## Results

The flow diagram of the inclusion of the study participants of the PROTECT trial has been shown previously [24]. In brief, among the 482 patients randomized (ipragliflozin, *N* = 241 and control, *N* = 241), 464 (ipragliflozin, *N* = 232 and control, *N* = 232) were included in the full analyses set of the PROTECT dataset. Detailed background demographics and characteristics have also been reported [24] and were well balanced between the allocation groups (Table 1). Overall, the median age was 68 years. Of the entire study population, 31.7% were women, and the median duration of T2DM was 8 years. Approximately 40% of the patients had a history of ASCVD, and 26 patients (5.6%) had a history of HF or cardiomyopathy. The proportion of patients who had been receiving diuretics at baseline was relatively small, and no significant difference was observed between both groups.

**Table 1:** Background demographic and clinical characteristics of the patients

Variable	Overall (*N* = 464)	Ipragliflozin (*N* = 232)	Control (*N* = 232)
Age (year)^a^, median (interquartile range)	68 (60, 73)	67 (60, 72)	68 (60, 73)
Sex			
Women, *n (%)*	147 (31.7)	71 (30.6)	76 (32.8)
Men, *n (%)*	317 (68.3)	161 (69.4)	156 (67.2)
BMI (kg/m^2^), median (interquartile range)	25.8 (23.4, 29.0)	25.3 (23.7, 28.9)	26.2 (23.2, 29.2)
Systolic BP (mm Hg)^a^, median (interquartile range)	130 (122, 141)	130 (120.5, 140)	130 (122, 141)
eGFR (mL/min/1.73 m^2^), median (interquartile range)	68.4 (58.9, 79.7)	68.8 (59.9, 77.2)	67.7 (58.3, 82.1)
Diabetes duration (year)^b^, median (interquartile range)	8.0 (3.9, 14.0)	8.5 (4.9, 14.0)	7.5 (3.7, 13.0)
HbA1c (%)^a^*, *median (interquartile range)	7.3 (6.8, 7.9)	7.2 (6.8, 7.9)	7.3 (6.7, 7.9)
Medical history			
Hypertension, *n (%)*	298 (64.2)	148 (63.8)	150 (64.7)
Dyslipidemia, *n (%)*	289 (62.3)	146 (62.9)	143 (61.6)
ASCVD, *n (%)*	182 (39.2)	95 (40.9)	87 (37.5)
HF and/or cardiomyopathy^c^, *n (%)*	26 (5.6)	12 (5.2)	14 (6.0)
Medication			
ACE inhibitor, *n (%)*	81 (17.5)	40 (17.2)	41 (17.7)
ARB, *n (%)*	208 (44.8)	95 (40.9)	113 (48.7)
Diuretics			
Loop, *n (%)*	35 (7.5)	21 (9.1)	14 (6.0)
Thiazide, *n (%)*	55 (11.9)	28 (12.1)	27 (11.6)
MRA, *n (%)*	39 (8.4)	20 (8.6)	19 (8.2)
Statin^a^, *n (%)*	309 (66.6)	153 (65.9)	156 (67.2)
Insulin, *n (%)*	20 (4.3)	9 (3.9)	11 (4.7)
Metformin^a^, *n (%)*	166 (35.8)	81 (34.9)	85 (36.6)
Sulfonylurea, *n (%)*	105 (22.6)	46 (19.8)	59 (25.4)
Thiazolidinedione, *n (%)*	46 (9.9)	24 (10.3)	22 (9.5)
DPP-4 inhibitor, *n (%)*	285 (61.4)	145 (62.5)	140 (60.3)
GLP-1 receptor agonist, *n (%)*	9 (1.9)	5 (2.2)	4 (1.7)

Data are presented as medians (interquartile ranges) or numbers (percentages)

ACE: angiotensin-converting enzyme; ARB: angiotensin receptor blocker; ASCVD: atherosclerotic cardiovascular disease; BP: blood pressure; BMI: body mass index; DPP-4: dipeptidyl peptidase-4; eGFR: estimated glomerular filtration rate; GLP-1: glucagon-like peptide-1; HF: heart failure; MRA: mineralocorticoid receptor antagonist

^a^Data at randomization

^b^Data were available for 183 patients in the ipragliflozin and control groups

^c^Investigator reported

**Fig. 1:**
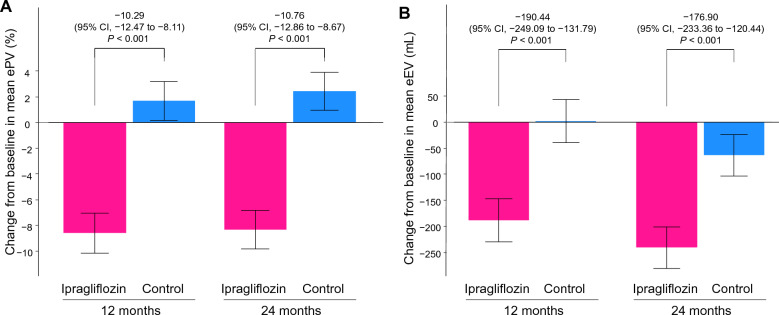
Comparisons of the changes over 24 months in the estimated fluid parameters (**A** ePV, **B** eEV) between the treatment groups. CI: confidence interval; eEV: estimated extracellular volume; ePV: estimated plasma volume

The baseline values and annual changes in ePV and eEV over 24 months are shown in Table 2. The baseline estimated fluid volume status as assessed by the ePV and eEV was similar between the treatment groups (a standardized mean difference of 0.076 for ePV and 0.075 for eEV, respectively). The reductions in the ePV and eEV at 12 and 24 months in the ipragliflozin group were significantly greater than those in the control group (all *P* < 0.001) (Fig. 1). In both groups, the changes in the ePV from 12 to 24 months were not obvious, whereas the eEV continued to decrease from 12 to 24 months (Table 3).

**Table 2:** Annual changes in the estimated fluid volume parameters over 24 months

Variables	Ipragliflozin (*N* = 232)		Control (*N* = 232)		Group difference* (95% CI)	*P*-value
	*n*	Mean ± SD	*n*	Mean ± SD		
ePV						
Baseline, mL^†^	223	2473 ± 328	220	2501 ± 389		
Change from baseline to 12 months, %	179	− 9.32 ± 8.65	191	1.79 ± 10.49	− 10.29 (− 12.47 to − 8.11)	< 0.001
Change from baseline to 24 months, %	206	− 8.66 ± 11.92	210	2.57 ± 13.11	− 10.76 (− 12.86 to − 8.67)	< 0.001
eEV						
Baseline, mL	229	14,021 ± 1505	227	14,142 ± 1702		
Change from baseline to 12 months, mL	192	− 196.43 ± 284.56	197	4.18 ± 244.97	− 190.44 (− 249.09 to − 131.79)	< 0.001
Change from baseline to 24 months, mL	217	− 241.90 ± 328.75	217	− 63.95 ± 334.48	− 176.90 (− 233.36 to − 120.44)	< 0.001

Data are expressed as means ± standard deviations

CI: confidence interval; eEV: estimated extracellular volume; ePV: estimated plasma volume

*Estimated by a longitudinal mixed-effects model for repeated measures

^†^The ePV at baseline was calculated using the Kaplan–Hakim formula

**Table 3:** Estimation of the changes in the parameters of interest from 12 to 24 months

Variables	Ipragliflozin (*N* = 232)			Control (*N* = 232)		
	*n*	Mean (95% CI)	*P-*value	*n*	Mean (95% CI)	*P-*value
Change in the ePV from 12 to 24 months, %	174	0.28 (− 1.20 to 1.75)	0.712	188	0.75 (− 0.68 to 2.18)	0.303
Change in the eEV from 12 to 24 months, mL	185	− 52.21 (− 91.72 to − 12.69)	0.010	192	− 65.75 (− 104.74 to − 26.76)	0.001
Change in hemoglobin from 12 to 24 months, g/dL	174	0.01 (− 0.11 to 0.13)	0.860	188	− 0.03 (− 0.15 to 0.09)	0.580
Change in hematocrit from 12 to 24 months, %	174	− 0.05 (− 0.41 to 0.32)	0.797	188	− 0.11 (− 0.46 to 0.24)	0.538
Change in body weight from 12 to 24 months, kg	187	− 0.53 (− 0.99 to − 0.07)	0.024	197	− 0.76 (− 1.21 to − 0.31)	0.001

Estimated by a mixed-effects model for repeated measures

CI: confidence interval; eEV: estimated extracellular volume; ePV: estimated plasma volume

**Fig. 2:**
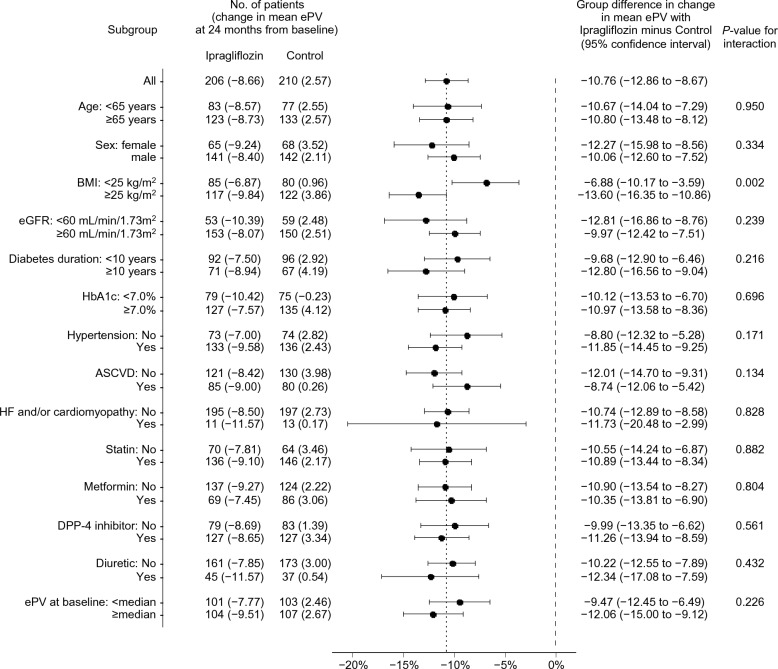
Subgroup analysis of the changes in the ePV from baseline to 24 months. ASCVD: atherosclerotic cardiovascular disease; BMI: body mass index; DPP-4: dipeptidyl peptidase-4; eGFR: estimated glomerular filtration rate; ePV: estimated plasma volume; HF: heart failure

**Fig. 3:**
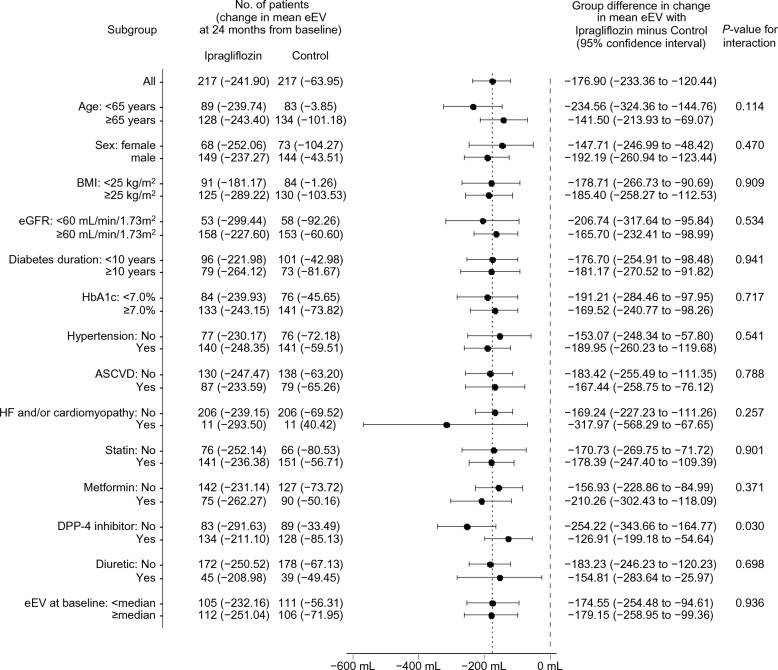
Subgroup analysis of the changes in the eEV from baseline to 24 months. *ASCVD, atherosclerotic cardiovascular disease*; BMI: body mass index; DPP-4: dipeptidyl peptidase-4; eEV: estimated extracellular volume; eGFR: estimated glomerular filtration rate; HF: heart failure

The effects of ipragliflozin on the estimated fluid volume parameters over 24 months were almost consistent across the subgroups examined, according to several background information [ePV (Fig. 2) and eEV (Fig. 3)]. All *P* values for the interactions, except for the subgroups according to the BMI category for ePV and DPP-4 inhibitor use for eEV, were > 0.1.

**Fig. 4:**
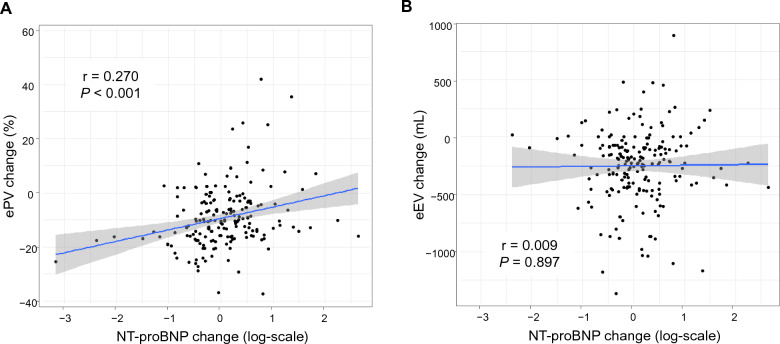
Scatterplots showing the correlations between the changes in the estimated fluid volume parameters (**A** ePV, **B** eEV) and log-scaled NT-proBNP concentration at 24 months. The mean regression line (blue line) and 95% confidence intervals (gray zone) are displayed. eEV: estimated extracellular volume; ePV: estimated plasma volume; NT-proBNP: N-terminal pro-brain natriuretic peptide

The geometric mean NT-proBNP concentration at baseline and 24 months in the control group was 62.33 pg/mL (95% CI 53.11 to 73.14 pg/mL) and 69.97 pg/mL (95% CI 59.47 to 82.34 pg/mL), respectively, and its proportional change from baseline to 24 months was 1.12 (95% CI 1.03 to 1.23; *P* = 0.011). Twenty-four months of ipragliflozin treatment did not affect the geometric mean NT-proBNP concentration (68.43 pg/mL [95% CI 58.30 to 80.34 pg/mL] at baseline and 73.64 pg/mL [95% CI 62.52 to 86.74 pg/mL] at 24 months and its proportional change [1.08; 95% CI 0.98 to 1.18; *P* = 0.113]). The group ratio (ipragliflozin vs. control) of the proportional changes in the geometric mean NT-proBNP concentration was 0.95 (95% CI 0.84 to 1.08; *P* = 0.434). In the ipragliflozin group, the change from baseline to 24 months in the log-transformed NT-proBNP concentration was modestly positively correlated with the corresponding change in the ePV (Fig. 4A), but not with that in the eEV (Fig. 4B).

The changes from baseline to 12 and 24 months in the parameters (i.e., hemoglobin, hematocrit, and body weight) incorporated into the formulas used to calculate the ePV and eEV are shown in Table 4. Significant group differences in the changes were observed from baseline to 12 and 24 months for each parameter. In both groups, the changes in hemoglobin and hematocrit from 12 to 24 months were not obvious, whereas body weight continued to decrease from 12 to 24 months (Table 3). Changes in other laboratory data, including glycemic parameters, from baseline to 24 months have been reported previously [24].

**Table 4:** Changes in the parameters incorporated into the formulas for calculating the ePV and eEV*

Variables	Ipragliflozin (*N* = 232)		Control (*N* = 232)		Group difference^†^ (95% CI)	*P*-value
	*n*	Mean ± SD	*n*	Mean ± SD		
Hemoglobin, g/dL						
Baseline	224	14.05 ± 1.54	220	14.05 ± 1.50		
Change from baseline to 12 months	179	0.79 ± 0.78	191	− 0.10 ± 0.82	0.83 (0.65 to 1.00)	< 0.001
Change from baseline to 24 months	206	0.77 ± 1.02	210	− 0.13 ± 1.04	0.87 (0.70 to 1.04)	< 0.001
Hematocrit, %						
Baseline	224	41.79 ± 4.08	220	41.90 ± 4.07		
Change from baseline to 12 months	179	2.69 ± 2.36	191	− 0.32 ± 2.53	2.85 (2.32 to 3.38)	< 0.001
Change from baseline to 24 months	206	2.55 ± 2.94	210	− 0.46 ± 3.05	2.91 (2.40 to 3.42)	< 0.001
Body weight, kg						
Baseline	231	68.82 ± 13.17	232	70.26 ± 15.61		
Change from baseline to 12 months	194	− 2.24 ± 3.23	202	− 0.01 ± 2.86	− 2.14 (− 2.82 to − 1.47)	< 0.001
Change from baseline to 24 months	219	− 2.69 ± 3.75	222	− 0.77 ± 3.97	− 1.91 (− 2.56 to − 1.26)	< 0.001

CI: confidence interval; eEV: estimated extracellular volume; ePV: estimated plasma volume; SD: standard deviation

*Height was excluded, because the eEV at each timepoint was calculated using the height at baseline

^†^Estimated by a longitudinal mixed-effects model for repeated measures

## Discussion

Accumulated cardiovascular and renal outcomes trials showed that SGLT2 inhibitors reduced the risk of cardiovascular and renal events, particularly HF-related events, in various patient populations, irrespective of diabetes and other clinical status [6–8]. However, the precise mechanisms underlying such clinical benefits of SGLT2 inhibitors remain to be fully understood. This fact may result from multifaceted subsequent effects, such as hemodynamic and metabolic actions, following natriuresis and glycosuria primarily caused by SGLT2 inhibition [26, 27], accordingly evoking changes in diverse clinical parameters. This also makes it difficult to clinically identify the key parameters to monitor the cardiovascular benefits of SGLT2 inhibitor therapy [9]. In this context, previous several mediation analyses using data obtained from some cardiovascular outcome trials with SGLT2 inhibitors have shown that erythrocyte concentration and changes in the plasma volume-related markers, independent of glycemic parameters, were the strongest mediators of the risk reduction in the cardiorenal events of SGLT2 inhibitors [28–31]. Therefore, those hemodynamic markers may be potential clinical markers to monitor the cardiorenal benefits after the initiation of SGLT2 inhibitor therapy.

To date, several clinical studies have assessed the impact of SGLT2 inhibitor therapy on the estimated plasma volume status over several weeks [10–15]. Dekkers et al. [11] first reported that a SGLT2 inhibitor (dapagliflozin) gradually reduced the ePV as assessed using the Strauss formula until 12 weeks and that the ePV status plateaued for the next 12 weeks in patients with T2DM. After that, the ePV has been reported to decrease gradually after the initiation of a SGLT2 inhibitor (empagliflozin) and sustain for 12 weeks in patients with HF with reduced ejection fraction [12] and for 1 week in inpatients with T2DM and acute decompensated HF [13]. Furthermore, we demonstrated that SGLT2 inhibitor therapy for 24 weeks significantly decreased the ePV and eEV as assessed based on the body surface area of patients with T2DM and cardiovascular disease (CVD) [14] and in patients with T2DM and chronic HF [15]. Matsuba et al. [32] also revealed that canagliflozin decreased the ePV calculated using the Kaplan formula and the impedance method-based extracellular water composition for 12 months. However, no study has addressed the longer-term effects of SGLT2 inhibitors on fluid volume parameters. Thus, our findings may expand the previous knowledge about the gradual short-term reduction in the estimated fluid volume parameters after the initiation of SGLT2 inhibitors and highlight the chronic regulation of fluid volume homeostasis by SGLT2 inhibitors.

The fluid volume status evaluated in this analysis was alternatively estimated using the existing formulas. Accordingly, the estimated values likely reflected, at least in part, the dynamics of the parameters incorporated into the formulas. Particularly, the trajectories of erythrocytic markers (i.e., hemoglobin and hematocrit) seemed to be linked to that of the ePV in our study. Several studies have consistently shown that a SGLT2 inhibitor therapy enhanced hematopoiesis in a broad range of patient populations, and this phenomenon is not merely caused by hemoconcentration [19, 33, 34]. Rather, the erythrogenesis through SGLT2 inhibition is involved in the promoted erythropoietin production, contributing to its cardiorenal benefits via several mechanisms, such as the amelioration of oxygen delivery and intrarenal hypoxia [35–38]. Furthermore, in this analysis, the chronic reduction in the ePV, but not the eEV, in patients treated with ipragliflozin was associated with a decrease in the log-scaled NT-proBNP concentration, suggesting an alleviation of left ventricular wall stress. The findings were also observed in our previous analysis using data from the EMBLEM trial, exploratorily investigating the effects of empagliflozin administered for 24 weeks, compared with placebo, on the ePV and eEV in patients with T2DM and established CVD [14]. Collectively, the ePV response to SGLT2 inhibition chronically represents not only the fluid volume dynamics but also the erythropoiesis reaction, which could be a reliable surrogate marker for the long-term monitoring of the cardiorenal benefits of SGLT2 inhibitor therapy [5].

In this analysis, the burdens of ipragliflozin-induced reduction in the ePV, representative of the circulating volume, did not alter from 12 to 24 months, whereas those in the eEV, representative of the non-intracellular volume, further augmented during that interval (Table 3). Interestingly, similar trends were also observed in our previous studies over 24 weeks, where the ePV stopped falling and plateaued after 12 weeks of SGLT2 inhibitor administration, whereas the eEV continued falling over 24 weeks [14, 15]. Given the SGLT2 inhibitor-mediated modest impact on the circulating volume and preferential removal of interstitial fluid volume [3, 4], the difference observed in the time course of the ePV and eEV would be reasonable. At the chronic phase of SGLT2 inhibitor therapy, the reduced but preserved ePV will avoid the loss of tissue/organ perfusion, and the continued eEV removal will regulate fluid volume imbalance and mitigate congestion, potentially supporting, at least in part, the aforementioned clinical benefits of SGLT2 inhibitors, particularly for HF.

This work has several limitations, which are largely inherit from our previous studies examining the similar endpoints to these analyses [14, 15]. First, the fluid volumes were estimated using the formulas used in the aforementioned studies, but not measured directly. Although a moderate correlation between changes in the estimated and directly measured fluid volumes has been reported previously [11, 39], validating our findings using gold-standard radiolabeled or impedance methods would be required [40, 41]. Furthermore, we cannot exclude a possibility that the promoted erythropoiesis and body weight loss induced by ipragliflozin treatment might have biased each formula used, independently of estimation of plasma and extracellular volumes. Second, although this was a prespecified secondary analysis of the PROTECT trial, information on the changes in the ePV and eEV after the initiation of ipragliflozin at follow-up visits other than 12 and 24 months was unavailable. Additionally, because the PROTECT trial was not designed to assess the effects of ipragliflozin on the incidence of cardiorenal events, we cannot show the relationship between the changes in the estimated fluid volume parameters analyzed and those clinical events. Third, the PROTECT trial was an open-label design study, which potentially caused unexpected bias toward the endpoints measured in this study. Particularly, the investigators’ medication selection during the follow-up period can affect them. However, in this study, only minor changes in the use of several medications, which potentially influence the fluid volume and body weight, were reported at 24 months (Additional fle 1).

In summary, ipragliflozin treatment, compared with the standard care for T2DM, reduced two types of estimated fluid volume parameters in patients with T2DM, and the effect was maintained for at least 24 months. Our findings suggest that SGLT2 inhibitor treatment regulates clinical parameters incorporated into the calculating formulas analyzed and consequently fluid volume status in the long-term. Therefore, those estimated parameters may be useful in monitoring the longitudinal clinical benefits of SGLT2 inhibitors.

## Supplementary Information

**Additional file 1.** Relevant medication uses at 24 months.

## Data Availability

The data are available upon reasonable request from the researchers who submit a detailed proposal outlining their intended use of the data and after approval by the principal investigators and the steering committee of the PROTECT trial. Inquiries are to be addressed to the corresponding author (or study secretariat: substudy_protect@clin-med.org).

## Abbreviations

ASCVD: Atherosclerotic cardiovascular disease
BMI: Body mass index
CI: Confidence interval
CVD: Cardiovascular disease
eEV: Estimated extracellular volume
eGFR: Estimated glomerular filtration rate
ePV: Estimated plasma volume
HF: Heart failure
IMT: Intima-media thickness
NT-proBNP: N-terminal pro-brain natriuretic peptide
SGLT2: Sodium-glucose co-transporter 2
T2DM: Type 2 diabetes mellitus
