# Supplementary material for: Long-term observation of estimated fluid volume reduction after the initiation of ipragliflozin in patients with type 2 diabetes mellitus: a sub-analysis from a randomized controlled trial (PROTECT)
Source: Diabetol Metab Syndr. 2023 Jul 7;15:152. doi: 10.1186/s13098-023-01129-3 (PMC10326942; doi:10.1186/s13098-023-01129-3)
Supplement: Supplementary file 1 — Additional file 1. Relevant medication uses at 24 months. [file 13098_2023_1129_MOESM1_ESM.docx]

**Additional File 1.** Relevant medication uses at 24 months.

| **Medication** | **Ipragliflozin**  **(*N* = 231)** | **Control**  **(*N* = 227)** |
| --- | --- | --- |
| Diuretics |  |  |
| Loop | 22 (9.5)) | 14 (6.2) |
| Thiazide | 27 (11.7) | 26 (11.5) |
| MRA | 19 (8.2) | 20 (8.8) |
| Insulin | 11 (4.8) | 11 (4.8) |
| Metformin | 81 (35.1) | 108 (47.6) |
| Sulfonylurea | 40 (17.3) | 57 (25.1) |
| Thiazolidinedione | 20 (8.7) | 21 (9.3) |
| GLP-1 receptor agonist | 6 (2.6) | 8 (3.5) |

Data are shown as numbers (%) among patients for whom drug information was available at 24 months.

*GLP-1, glucagon-like peptide-1; MRA, mineralocorticoid receptor antagonist.*
